# Keap1-Kelch-targeting protein–protein interaction inhibitors, but not reversibly-binding electrophiles, increase the thermostability of Keap1 in the cellular environment

**DOI:** 10.1039/d6cb00045b

**Published:** 2026-04-07

**Authors:** Sharadha Dayalan Naidu, Dina Dikovskaya, Jasmine M. Walker, Charlotte Lim Jia Yee, Annamarie J. Cafferkey, Manaka Tatsuno, Jialin Feng, Terry W. Moore, Tatum Johnson, Tadashi Honda, Geoff Wells, Takafumi Suzuki, Masayuki Yamamoto, Albena T. Dinkova-Kostova

**Affiliations:** a Jacqui Wood Cancer Centre, Division of Cancer Research, School of Medicine, University of Dundee Dundee UK s.z.dayalannaidu@dundee.ac.uk A.DinkovaKostova@dundee.ac.uk; b Peninsula Medical School, Faculty of Health, University of Plymouth Plymouth UK; c Department of Biochemistry and Molecular Biology, Tohoku Medical Megabank Organization, Tohoku University Sendai Japan; d Department of Pharmaceutical Sciences, College of Pharmacy, University of Illinois Chicago Chicago IL USA; e Department of Chemistry and Institute of Chemical Biology & Drug Discovery, Stony Brook University Stony Brook NY USA; f UCL School of Pharmacy, University College London 29/39 Brunswick Square London UK; g Department of Physiology, Pharmacology and Therapeutics, Johns Hopkins University School of Medicine Baltimore MD USA; h Department of Medicine, Johns Hopkins University School of Medicine Baltimore MD USA

## Abstract

The Kelch-like ECH-associated protein 1/nuclear factor erythroid 2-p45-related factor 2 (Keap1/Nrf2) partnership orchestrates the cellular defence against oxidative, inflammatory and metabolic stress. Dysregulation of Nrf2 is involved in the pathogenesis of numerous chronic diseases. Under homeostatic conditions, Keap1 continuously targets Nrf2 for ubiquitination and degradation. When Keap1 is inactivated, Nrf2 accumulates and translocates to the nucleus, where it activates transcription of genes encoding cytoprotective proteins. There are two main types of Keap1 inhibitors, electrophiles and Keap1-Nrf2 protein–protein interaction (PPI) inhibitors. Using a quantitative fluorescence-based cellular thermal shift assay (CETSA), we investigated the ability of a panel of electrophilic Nrf2 activators and PPI inhibitors to bind to Keap1 in lysates and intact cells stably expressing Keap1-mCherry or free mCherry as controls. All PPI inhibitors tested caused an increase in the thermostability of Keap1-mCherry. Surprisingly however, electrophiles that bind covalently and reversibly to thiols did not. Moreover, treatment of intact cells with the double Michael acceptors bis(benzylidene)acetone and its hydroxylated derivative bis(2-hydroxybenzylidene)acetone caused a decrease in the thermostability of Keap1. Thus, in addition to confirming target engagement of Keap1-targeting PPI inhibitors in the cellular environment, the Keap1 fluorescence-based CETSA is capable of distinguishing between the mechanism of action of the two types of Nrf2 activators in the cellular environment, and has the potential for cost-effective, high-throughput applications.

## Abstract 2

Keap1-Kelch-targeting protein–protein interaction inhibitors (left) increase the thermostability of Keap1-mCherry, whereas double Michael acceptors (right) decrease the thermostability of Keap1-mCherry in the cellular environment.

## Graphical abstract

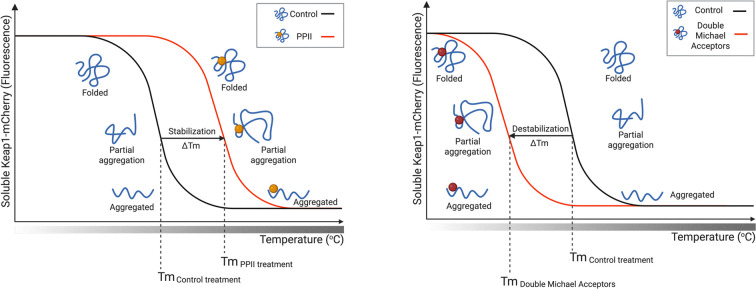

## Introduction

All living organisms are continuously exposed to potential sources of oxidative stress such as electrophiles and reactive oxygen and nitrogen species throughout their lifespan; these can be both endogenous from normal metabolic processes such as respiration and inflammation, or exogenous from environmental sources such as solar ultraviolet (UV) radiation, heavy metals, and pro-carcinogens. Redox signalling is essential for most biological processes; however, oxidative stress and persistent inflammation underlie the pathogenesis of essentially all chronic diseases.^1–3^

**Fig. 1:**
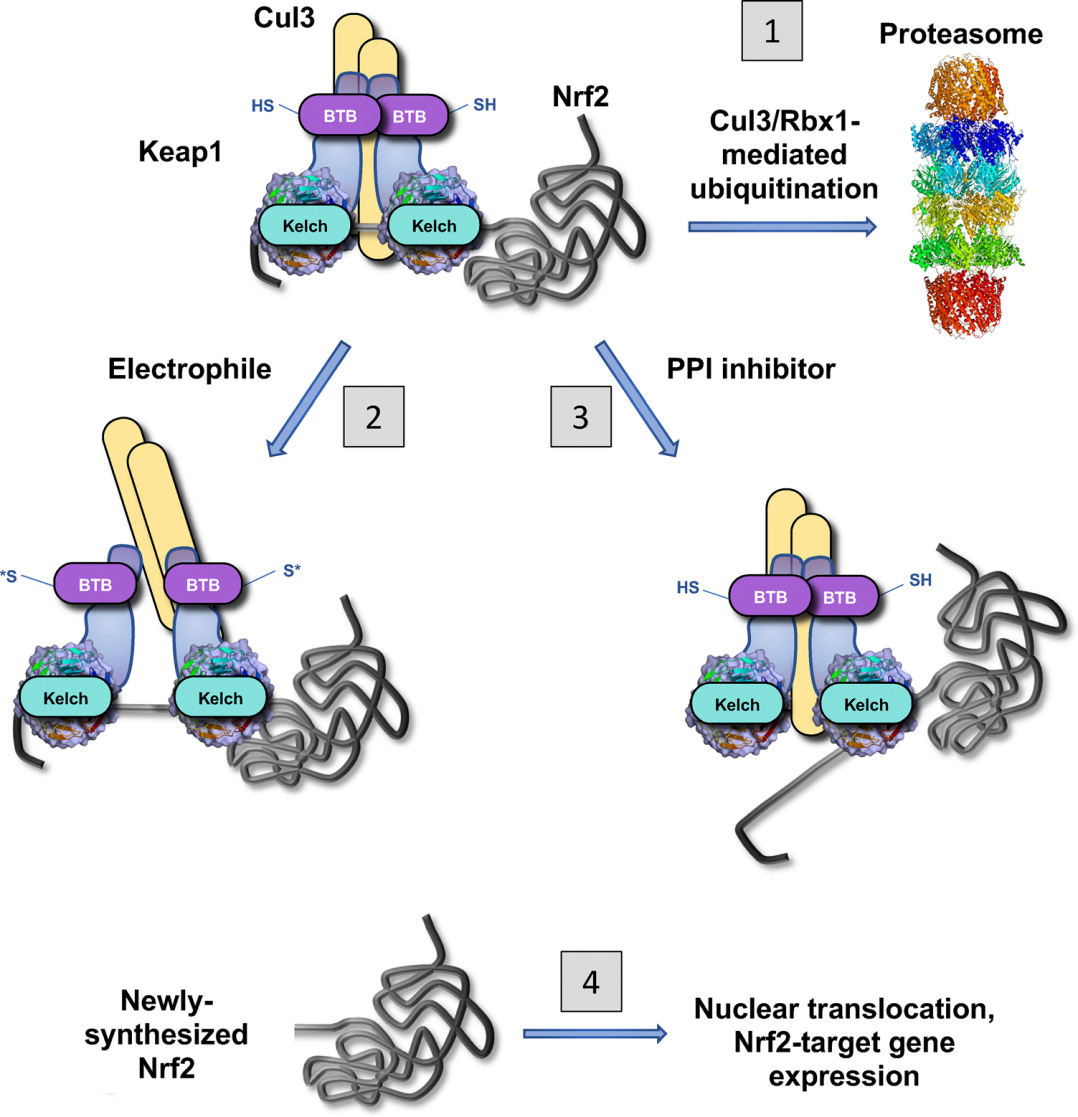
The Keap1-Nrf2 system. The E3 ubiquitin ligase substrate adaptor Keap1 targets transcription factor Nrf2 for Cul3/Rbx1-mediated ubiquitination and subsequent degradation through the proteasome (1). Electrophiles, many of which bind covalently to C151 in the BTB domain of Keap1 lower the affinity of Keap1 to Cul3, thus impairing the substrate adaptor function without disrupting the Keap1–Nrf2 interaction (2). Small-molecule Keap1–Nrf2 protein–protein interaction (PPI) inhibitors bind to the Kelch domain of Keap1, partially disrupting its interaction with Nrf2 (3). In both cases, newly-synthesized Nrf2 accumulates, undergoes nuclear translocation, and activates transcription of its target genes (4). SH = reduced cysteine; S* = modified cysteine.

In order to combat oxidative stress and restore the redox state to homeostasis, cells and organisms employ robust antioxidant defence systems. The transcription factor nuclear factor erythroid 2-p45-related factor 2 (Nrf2) is an essential component of the cellular antioxidant defences, by controlling the gene expression of numerous antioxidant enzymes, including NAD(P)H: quinone oxidoreductase 1 (NQO1) and heme oxygenase 1 (HO1), as well as enzymes that catalyse the biosynthesis of glutathione and its maintenance in the reduced (GSH) state.^4,5^ Although ubiquitously expressed, the levels of Nrf2 are maintained low due to continuous ubiquitination and subsequent proteasomal degradation of the transcription factor (Fig. 1). The principal negative regulator of Nrf2 is Kelch-like ECH-associated protein 1 (Keap1), a substrate adaptor protein for Cullin 3 (Cul3) RING-box protein 1 (Rbx1) E3 ubiquitin ligase.

The cytoprotective effect of Nrf2 activation have been clearly demonstrated in multiple experimental systems, as well as in humans, with many small molecule Nrf2 activators currently at various stages of drug development^6,7^ In addition, endogenously produced electrophiles activate Nrf2, which in turn prevents their potentially toxic accumulation and restores homeostasis by inducing the expression of genes encoding enzymes that catalyze detoxification reactions.^8^ Most Nrf2 activators (also known as inducers) are electrophiles or Keap1-Nrf2 protein–protein interaction (PPI) inhibitors.^7^ Electrophiles activate Nrf2 by chemically modifying cysteine sensors on the Keap1 dimer,^9^ consequently impairing its repressor function (Fig. 1). A very large number of structurally-diverse electrophiles have been shown to activate Nrf2 in cells and *in vivo*, and three compounds are currently licenced for medical use: the fumaric acid esters dimethyl fumarate (DMF, brand name TECFIDERA) and diroximel fumarate (brand name VUMERITY) for the treatment of relapsing remitting multiple sclerosis and chronic plaque psoriasis, and the cyanoenone triterpenoid RTA-408 (omaveloxolone; brand name SKYCLARYS) for patients with Friedreich's ataxia.^6,10^ Both DMF and RTA-408 react covalently with Cys151 located in the Broad complex, Tramtrack and Bric-à-Brac (BTB) domain of Keap1,^11–13^ the site of interaction with Cul 3. Within the Keap1 protein structure, Cys151 is surrounded by basic amino acids;^14^ with a pKa of 6.9, this cysteine is highly reactive at physiological pH, and through mainly hydrophobic interactions, it binds structurally diverse electrophiles, with a key hydrogen bond orienting the electrophilic carbon for a catalytic proximity effect.^15^ Multiple other cysteines in Keap1 can undergo oxidative or electrophilic modifications, including Cys226, Cys273, Cys288, Cys297, Cys613, Cys622 and Cys624, with a high degree of preference for specific compounds, which is referred to as the “cysteine code”.^14,16,17^ Thus, the anti-inflammatory cyclopentenone prostaglandin 15-deoxy-Δ^12,14^-prostaglandin J_2_ (15d-PGJ_2_) and the prototypical nitro fatty acid, nitro-oleic acid, react with Cys273 and Cys288;^18,19^ the preferences for specific cysteine sensors in Keap1 of other endogenous electrophilic metabolites has been recently reviewed.^8,20^ Notably, distinct from electrophiles, Keap1 uses a ‘fail-safe’ mechanism that allows any combination among Cys226, Cys613, and Cys622/Cys624 to form a disulfide bond, for sensing of the oxidant hydrogen peroxide (H_2_O_2_).^21^

In addition to DMF and diroximel fumarate, Cys151 in Keap1 is modified by other electrophilic compounds with immuno-modulatory activities, such as 4-octyl itaconate, a cell-permeable derivative of the anti-inflammatory metabolite itaconate, and the kynurenine metabolite kynurenine carboxyketoalkene (Kyn-CKA).^22,23^ A chemoproteomics screening-based drug discovery program aiming to identify Cys151-targeting Nrf2 activators for the treatment of autoimmune disorders, has led to the unexpected discovery of compounds that selectively bind to this cysteine, but rather than inhibition, their binding results in activation of Keap1.^24^ It was further found that in contrast to the Cys151-targeting electrophiles mentioned above, which decrease the interactions between Keap1 and Cul3^25^ and de-repress Nrf2, the electrophile VVD-065 which binds covalently and selectively to the same cysteine, promotes formation of the Keap1-Cul3 protein complex, thus acting as a molecular glue enhancing the repressor activity of Keap1 and facilitating Nrf2 degradation.^24,25^

In contrast to electrophiles, the non-electrophilic Nrf2 activators developed to date disrupt the protein–protein interactions between Keap1 and Nrf2 by binding non-covalently to the Kelch domain of Keap1, the site of interaction with Nrf2, and thus partially displacing Nrf2 from Keap1 (Fig. 1).^26,27^ Among the Keap1-Nrf2 PPI inhibitors are compounds of diverse core chemical structures, such as 1,2,3,4-tetrahydroisoquinolines, 1,4-diaminonaphthalenes, 3-phenylpropanoic acids, 1-phenylpyrazoles, 1,4-diphenyl-1,2,3-triazoles, and hydrazinecarbohydrazides,^28–30^ as well as binding modes.^31,32^ Most high-affinity Keap1-Nrf2 PPI inhibitors are relatively large, carboxylic acid-containing molecules – this is because of the large size and polarity of the Kelch domain, which harbours positively charged amino acids, including Arg380, Asn382, Arg415, and Arg483 that make key salt bridges with acidic residues in Nrf2.^33^ These properties pose limitations on the membrane permeability, metabolic stability and oral bioavailability of the compounds. Despite these challenges, the continuous efforts of many investigators have led to the development of several metabolically stable Keap1-Nrf2 PPI inhibitors with potencies approaching the potencies of electrophilic Keap1 inhibitors and demonstrated *in vivo* efficacy.^27^ The inhibition of Keap1 by either electrophiles or PPI inhibitors leads to accumulation of newly synthesized Nrf2 and activation of Nrf2-target gene expression (Fig. 1).

We previously developed a quantitative fluorescence-based cellular thermal shift assay (CETSA) in lysates of cells stably expressing Keap1-mCherry fusion protein (and their free mCherry-expressing counterparts as controls), and used it to demonstrate Keap1 target engagement by two Keap1-Nrf2 PPI inhibitors, *i.e.*, the isoquinoline PRL-295^27,34^ and the naphthalene bis-sulfonamide NG-284.^31^ The aim of the present study was to establish whether this assay can be used as a simple, fast, inexpensive and high-throughput method to monitor target engagement of any Nrf2 activator that binds to Keap1, in the context of the cellular environment (cell lysates or intact cells). Using representative Keap1-Nrf2 PPI inhibitors and lysates from two different Keap1-mCherry-expressing cell lines (and their corresponding free mCherry-expressing counterparts), we show that such compounds, all of which bind to Keap1-Kelch non-covalently, cause an increase in the thermostability of Keap1-mCherry, but not free mCherry, in cell lysates and intact cells. Unexpectedly however, thermostabilization of Keap1-mCherry was not observed upon exposure to structurally diverse electrophilic Keap1 inhibitors. Furthermore, upon treatment of intact cells, the double Michael acceptors bis(benzylidene)acetone (DBA) or its hydroxylated derivative bis(2-hydroxybenzylidene)acetone (HBB2), which have two symmetric electrophilic centers, decreased the thermostability of the fusion protein. Additionally, treatment with DBA caused formation of reducing agent-resistant higher molecular weight Keap1 species that involve Cys151 and could be responsible for the observed decrease in the thermostability of Keap1.

## Results

### Generation of inducible Keap1-mCherry-expressing U2OS and HeLa cell lines

**Fig. 2:**
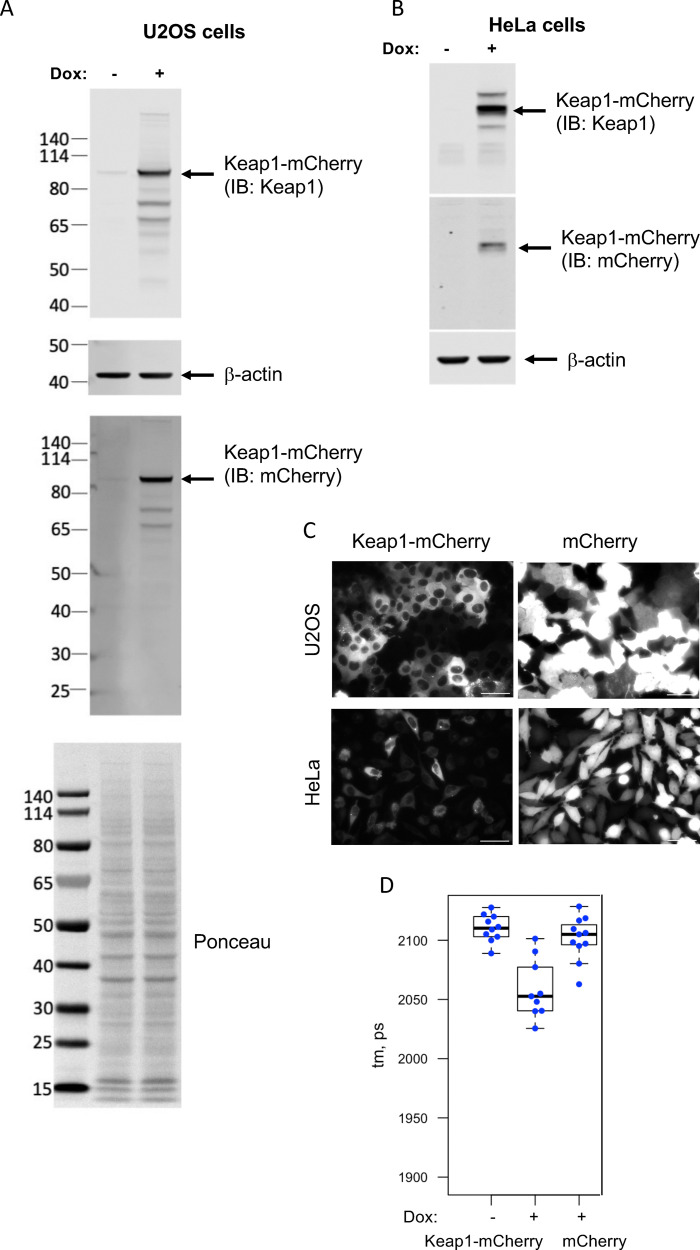
Inducible expression of Keap1-mCherry in U2OS and HeLa cells. (A) and (B) U2OS-FRT-TO (A) and HeLa-FRT-TO (B) cells with integrated Keap1-12fl-mCherry constructs were treated for 2 days with Dox or left untreated, and the lysates were immunoblotted for Keap1, mCherry and β-actin. Ponceau S staining prior immunoblotting shows equal loading. (C) mCherry fluorescence imaging of live Dox-treated U2OS-FRT-TO (top) or HeLa-FRT-TO (bottom) cells with integrated Keap1-12fl-mCherry (left) or mCherry (right). Scale bar 50 µm. (D) Dox-treated or untreated HeLa-FRT-TO with integrated Keap1-12-mCherry were transfected with sfGFP-Nrf2 and the interaction between sfGFP-Nrf2 and Keap1-mCherry in cytoplasmic areas was measured by FRET-FLIM assay. As a control, the same measurements were performed in sfGFP-Nrf2 transfected Dox-induced HeLa-FRT-TO with integrated mCherry. Fluorescence lifetime (tm) of sfGFP in individual cells is shown as dots, with boxplot outlining range between 25 and 75 percentiles, the thick line showing median, and the whiskers show minimum and maximum values excluding outliers.

The Flp-In™ T-REx™ system was employed to generate human U2OS and HeLa cell lines stably expressing free mCherry or a Keap1-mCherry fusion protein under the transcriptional control of a doxycycline (Dox)-inducible promoter. The fusion protein was based on a previously optimized Keap1-mCherry construct (Keap1-12fl-mCherry),^35,36^ which includes a 12-amino-acid flexible linker to allow for the independent, correct folding of the two proteins. The inducible expression of the Keap1-mCherry fusion protein was confirmed by immunoblotting of whole cell lysates using Keap1 and mCherry antibodies (Fig. 2A and B) and fluorescence microscopy (Fig. 2C). Importantly, Dox-inducible Keap1-mCherry is functional and forms a complex with overexpressed sfGFP-Nrf2, as revealed by Förster resonance energy transfer (FRET) between sfGFP and mCherry, shortening the fluorescence lifetime (*t*_m_) of the sfGFP donor fluorophore (Fig. 2D). By contrast, there is no difference in the fluorescence lifetime of sfGFP-Nrf2 between non-induced Keap1-mCherry-expressing and Dox-induced free mCherry-expressing cells.

### Keap1-targeting PPI inhibitors increase the thermostability of Keap1

**Fig. 3:**
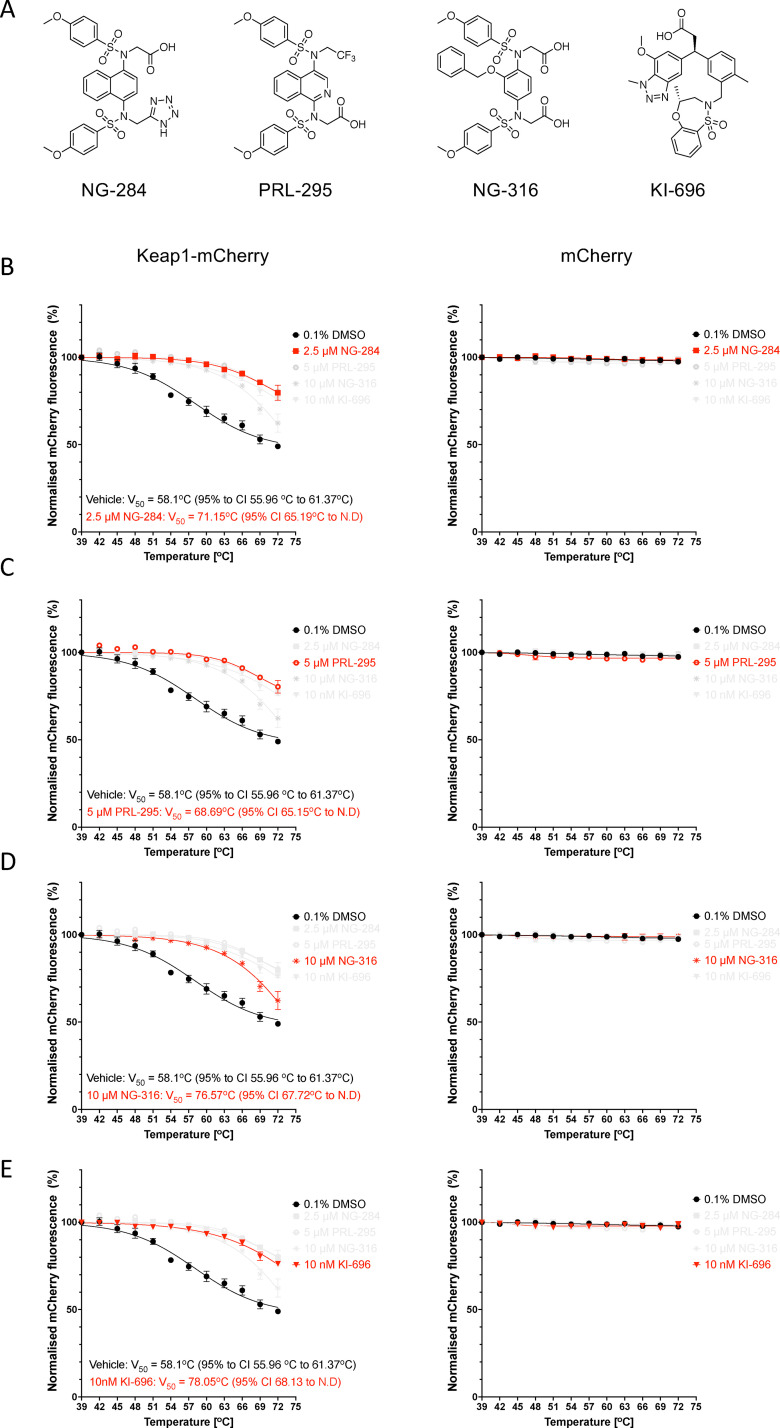
Keap1-mCherry is stabilized by incubation of HeLa cell lysates with Keap1-Nrf2 protein–protein interaction inhibitors. (A) Chemical structures of the PPI inhibitors used in this study. (B)–(E) Representative temperature-induced aggregation curves of Keap1-mCherry and mCherry following a 1-h incubation of lysates from Keap1-mCherry- or mCherry-expressing HeLa cells with 2.5 µM NG-284 (B), 5 µM PRL-295 (C), 10 µM NG-316 (D), 10 nM KI-696 (E) (red symbols) or vehicle (0.1% DMSO, black symbols). The mCherry fluorescence intensity was measured in soluble fractions of lysates heated to 39–72 °C and normalized to that of the soluble fraction of DMSO-treated lysate heated to 39 °C.

The first Keap1-Nrf2 PPI inhibitor tested was NG-284 (Fig. 3A). This compound is a tetrazole-containing naphthalene bis-sulfonamide with a high binding affinity for Keap1-Kelch (*K*_d_ < 1 nM) *in vitro*, which inhibits the Keap1-Nrf2 protein–protein interaction, and induces the expression of Nrf2 target genes in human and mouse cells,^31^ although the latter is observed at micromolar concentrations, most likely due to poor cell permeability. Similar to our previous observations in lysates from U2OS cells,^31^ one-hour incubation of lysates from HeLa cells with NG-284, increased the thermostability of Keap1-mCherry (Fig. 3B). By contrast, the treatment did not affect the fluorescence of free mCherry, which remained stable throughout this temperature range.

Next, we tested PRL-295 (Fig. 3A), a metabolically stable isoquinoline which disrupts the interactions between Keap1 and Nrf2 in cells and *in vitro*,^26,27^ and has been recently shown to promote wound healing in human keratinocytes and diabetic mice.^37^ We have previously found that PRL-295 increases the thermostability of endogenous Keap1 in cells as well as *in vivo*, in the mouse liver.^27,34^ Consistent with these results, incubation of Keap1-mCherry-expressing HeLa cell lysates with PRL-295 increased the thermostability of Keap1-mCherry in the soluble fraction of these lysates (Fig. 3C). By contrast, PRL-295 did not cause any change in the thermostability of mCherry, indicating that this compound is targeting the Keap1 portion of the Keap1-mCherry fusion protein.

We then tested the phenyl bis-sulfonamide Keap1-Nrf2 PPI inhibitor NG-316 (Fig. 3A), which binds to Keap1 in a distinct “peptidomimetic” conformation resembling the Keap1-Nrf2 ETGE peptide complex.^32^ We had previously shown that this compound increases the thermostability of endogenous Keap1 in lysates from HL-60 cells.^32^ In close agreement, we observed an increase in the thermostability of the Keap1-mCherry fusion protein (Fig. 3D) when lysates from Keap1-mCherry-expressing HeLa cells were incubated with NG-316, and no change in the thermostability of free mCherry.

Finally, we tested KI-696 (Fig. 3A), a benzotriazole derivative that was designed to target the interaction between Keap1 and Nrf2 using a fragment-based approach.^38^ This compound combines a high affinity for Keap1-Kelch with drug-like physicochemical properties and thus has a high potency as an Nrf2 activator both *in vitro* and *in vivo*. KI-696 has been shown to ameliorate ozone-mediated pulmonary inflammation in rats^38^ and to sensitize tumours to glucose-6-phosphate dehydrogenase (G6PD) inhibition in mice.^39^ More recently, KI-696 was employed as a recruitment handle for both Keap1-mediated- and Keap1-targeted protein degradation through proteolysis-targeting chimeras (PROTACs).^40–42^ Like the PPI inhibitors tested above, one-hour incubation with KI-696 of HeLa cell lysates expressing Keap1-mCherry-, but not free mCherry, increased the thermostability of Keap1 (Fig. 3E).

**Fig. 4:**
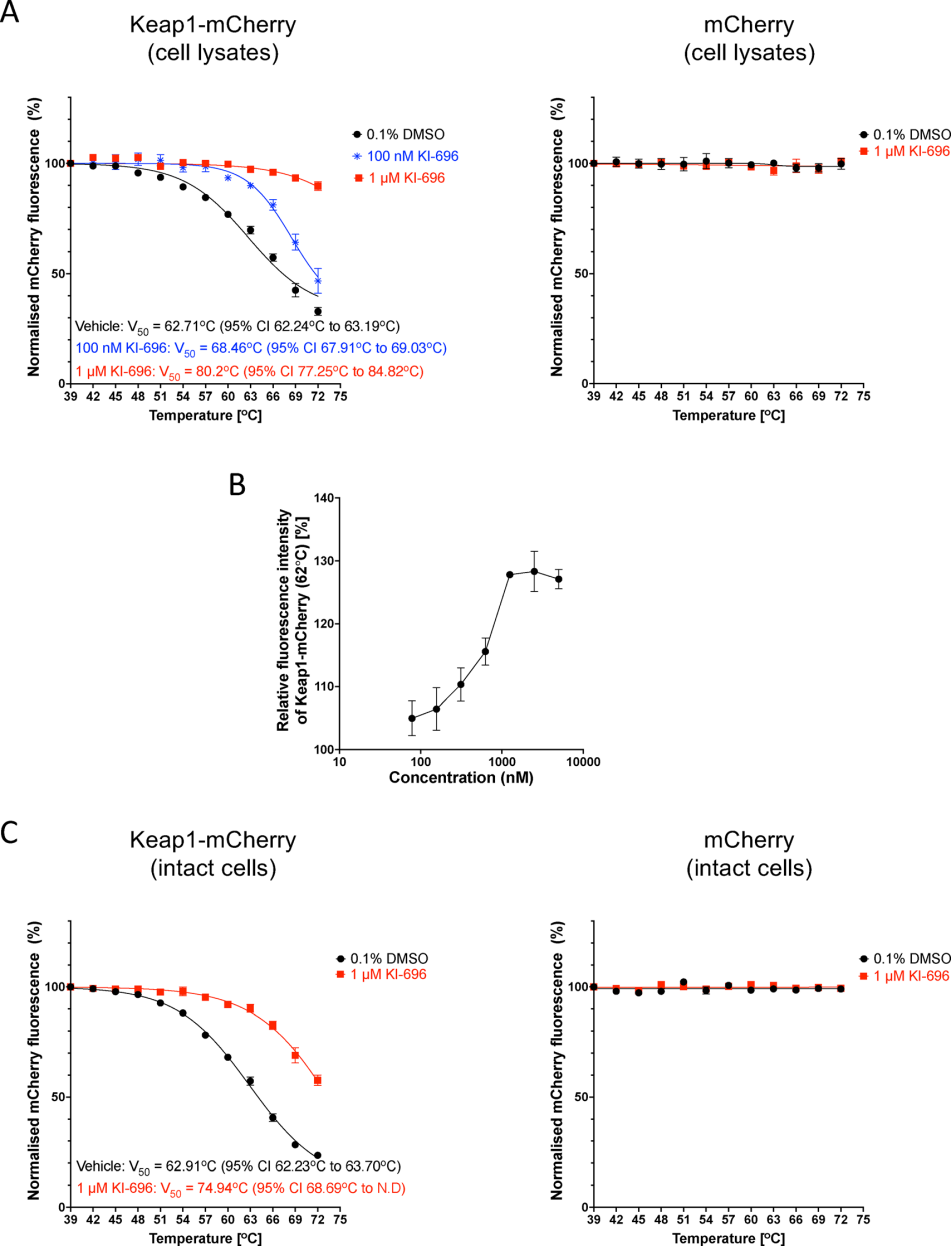
Keap1-mCherry is stabilized by incubation of U2OS cell lysates or intact cells with the Keap1-Nrf2 protein–protein interaction inhibitors KI-696. (A) Representative temperature-induced aggregation curves of Keap1-mCherry and mCherry following a 1-h incubation with 1 µM KI-696 (red symbols), 100 nM KI-696 (blue symbols) or vehicle (0.1% DMSO, black symbols) of cell lysates of Keap1-mCherry- or mCherry expressing U2OS cells. The mCherry fluorescence intensity was measured in soluble fractions of lysates heated to 39–72 °C and normalized to that of the soluble fraction of DMSO-treated lysate heated to 39 °C. (B) Isothermal dose–response (ITDR) curve showing concentration-dependent stabilization of Keap1-mCherry by KI-696. Lysates of cells expressing Keap1-mCherry were incubated with KI-696, at the indicated concentrations, for 1 h at 37 °C, then heated at 62 °C for 3 min, and the Keap1-mCherry fluorescence intensity was measured in the soluble fraction after removal of the aggregated material. (C) Representative temperature-induced aggregation curves of Keap1-mCherry and mCherry following a 3-h incubation with 1 µM KI-696 (red symbols) or vehicle (0.1% DMSO, black symbols) of intact Keap1-mCherry- or mCherry expressing U2OS cells, which were subsequently lysed. The mCherry fluorescence intensity was measured in soluble fractions of lysates heated to 39 °C–72 °C and normalized to that of the soluble fraction of DMSO-treated lysate heated to 39 °C.

Broadly similar results were obtained using lysates from U2OS cells, although in comparison with the Keap1-mCherry-expressing HeLa cells, the fluorescence intensity of mCherry in the Keap1-mCherry-expressing U2OS cells was higher (see Fig. 2C), whereas the thermostability of the fusion protein appeared lower. Following one-hour incubation and thermal denaturation of lysates from Keap1-mCherry- or mCherry-expressing U2OS cells, KI-696 caused a concentration-dependent increase in the thermostability of Keap1-mCherry, whereas the thermostability of free mCherry was not altered (Fig. 4A and B). The thermostability of Keap1-mCherry also increased when intact cells were treated with KI-696 (Fig. 4C). Notably, at the same concentration (1 µM), KI-696 induced a greater shift in the thermostability of Keap1-mCherry upon treatment of cell lysates in comparison with intact cells, consistent with direct access to the fusion protein without the need to cross the cell membrane. Together, these experiments firmly establish that PPI inhibitors that bind to the Kelch domain of Keap1 cause thermostabilization of Keap1, confirming engagement of the Keap1 protein target in the context of the cellular environment.

### Electrophilic Nrf2 activators do not increase the thermostability of Keap1-mCherry

**Fig. 5:**
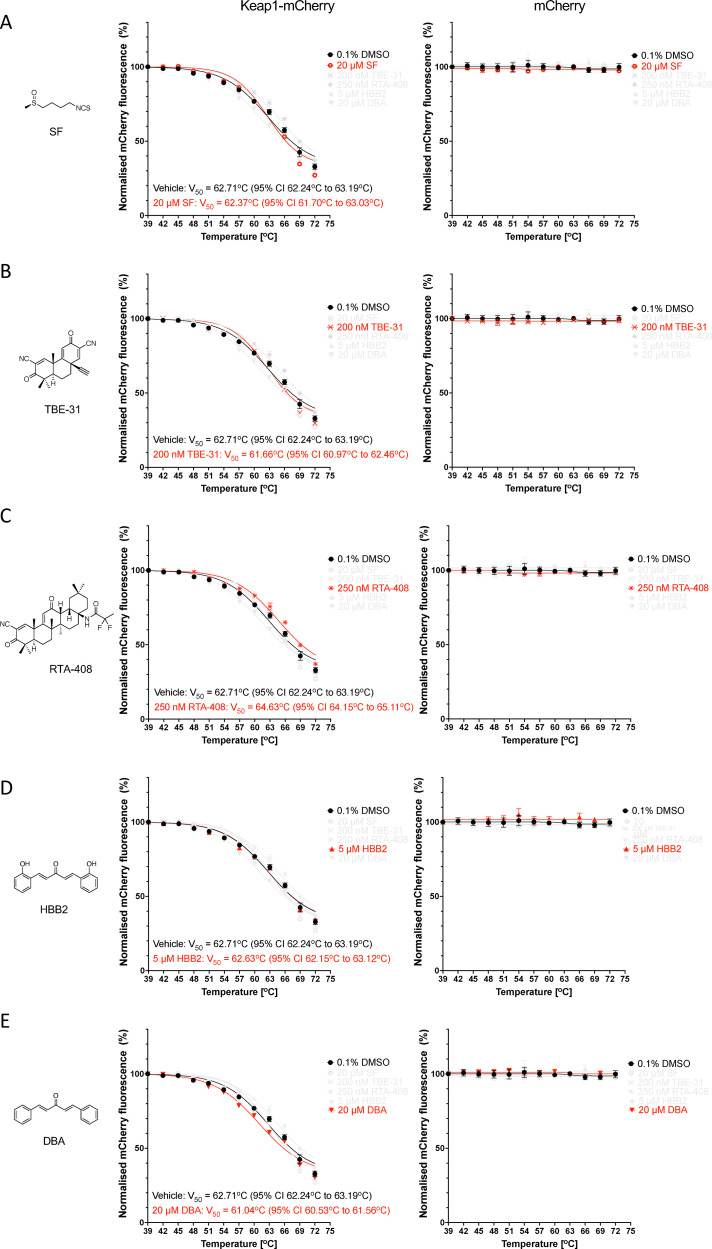
Keap1-mCherry is not stabilized by electrophilic Keap1 inhibitors in U2OS cell lysates. Representative temperature-induced aggregation curves of Keap1-mCherry or mCherry following a 1-h incubation of lysates from Keap1-mCherry- or mCherry-expressing U2OS cells with 20 µM sulforaphane, SF (A), 200 nM TBE-31 (B), 250 nM RTA-408 (C), 5 µM HBB2 (D), 20 µM DBA (E) (red symbols) or vehicle (0.1% DMSO, black symbols). The mCherry fluorescence intensity was measured in soluble fractions of lysates heated to 39–72 °C, normalized to that of the soluble fraction of DMSO-treated lysate heated to 39 °C.

Since the fluorescence of Keap1-mCherry was higher in lysates from Keap1-mCherry-expressing U2OS cells in comparison with their Keap1-mCherry-expressing HeLa counterparts (Fig. 2C), we used U2OS cells for our next experiments. Electrophilic Nrf2 activators bind covalently to cysteine sensors in Keap1, with Cys151 in the BTB domain being particularly reactive due to its proximity to basic amino acids within the structure of this domain.^14^ The reaction of Cys151 with structurally diverse Nrf2 activators is catalytic, with a key hydrogen bond orienting their electrophilic carbons within ∼3–5 Å of Cys151 for a catalytic proximity effect.^15^ Thus, we next tested the possibility that sulforaphane (SF), a classical electrophilic Nrf2 activator which targets Cys151,^43^ alters the thermostability of Keap1. Surprisingly, and in stark contrast to the PPI inhibitors, no thermal shift of the Keap1-mCherry fusion protein was observed when lysates from Keap1-mCherry-expressing (or free mCherry-expressing) U2OS cells were incubated with SF (Fig. 5A). Although unexpected, the above finding suggested that the fluorescence-based CETSA could potentially provide means to distinguish the mechanism of action between electrophiles and PPI inhibitors. Thus, we next asked whether other well-established, structurally diverse electrophilic Nrf2 activators may affect the thermostability of Keap1-mCherry.

Like SF, incubation of U2OS cell lysates with the Cys151-targeting tricyclic cyanoenone TBE-31^12^ did not affect the thermostability of Keap1-mCherry (Fig. 5B). TBE-31 is a highly potent, orally bioavailable Nrf2 activator,^44^ which is suitable for chronic administration,^45^ and has shown protective effects in many animal models of human disease, including aflatoxin-induced hepatocarcinogenesis, UV radiation-induced cutaneous carcinogenesis, and high-fat and fructose diet-induced steatohepatitis and hepatic fibrosis.^46–48^ Similar to its tricyclic derivative, the pentacyclic cyanoenone RTA-408 (omaveloxolone), which is currently used for the treatment of Friedreich's ataxia and also requires Cys151 in Keap1 to activate Nrf2,^13^ did not cause an appreciable change in the thermostability of Keap1-mCherry or free mCherry (Fig. 5C).

Next, we tested the double Michael acceptor bis(2-hydroxybenzylidene)acetone (HBB2) (Fig. 5D), which reacts readily with sulfhydryl groups and activates Nrf2,^49,50^ and the related non-hydroxylated bis(benzylidene)acetone (DBA) (Fig. 5E). Although less potent than its hydroxylated analog HBB2, DBA is an electrophile that also reacts with thiols and activates Nrf2-mediated gene expression.^49^ Similar to the results for the electrophiles described above, no increase in the thermal stability of Keap1-mCherry was observed when U2OS cell lysates were incubated with either HBB2 (Fig. 5D) or DBA (Fig. 5E).

### The double Michael acceptor bis(benzylidene)acetone and its hydroxylated derivative bis(2-hydroxybenzylidene)acetone decrease the thermostability of Keap1-mCherry in cells

**Fig. 6:**
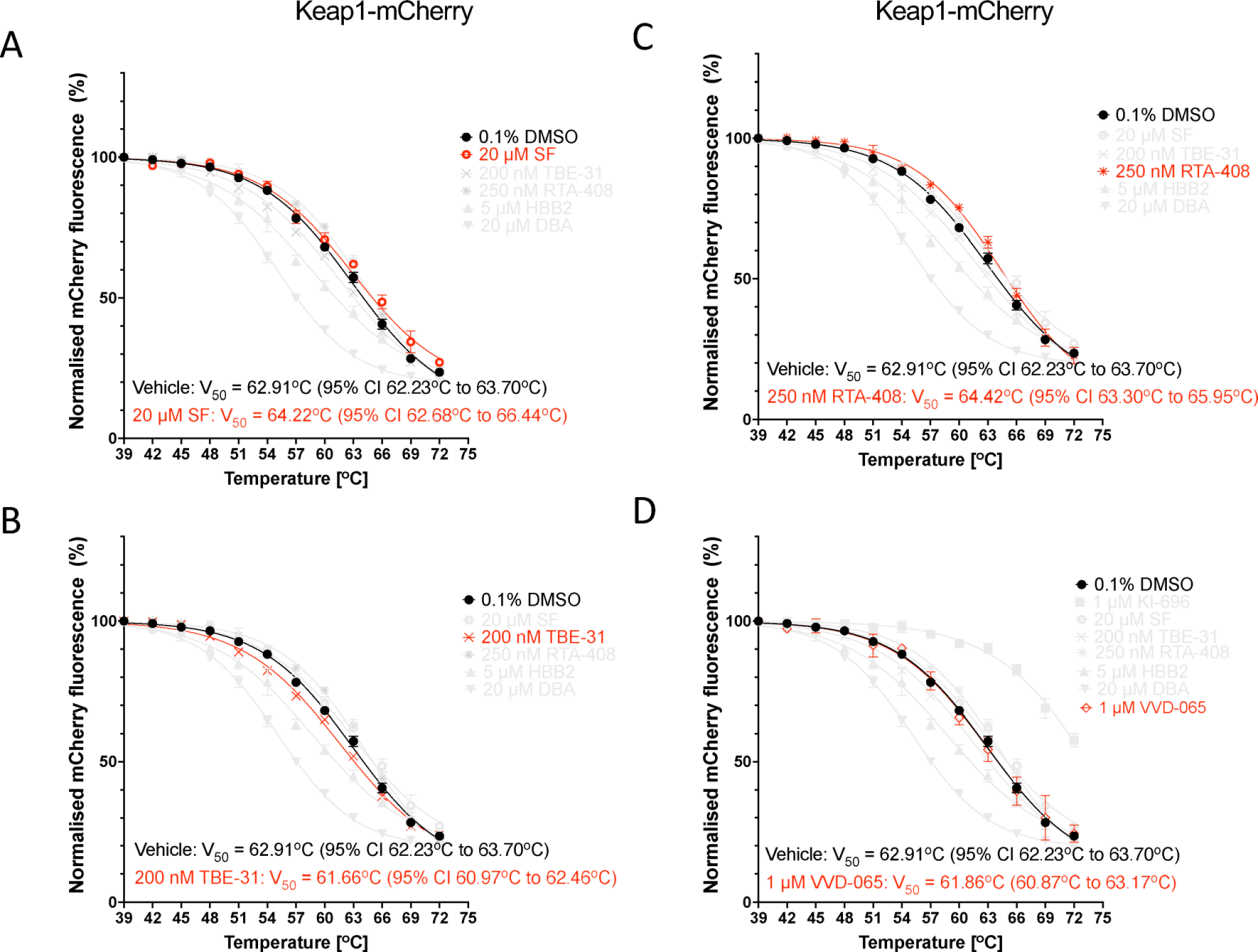
Keap1-mCherry is not stabilized by electrophilic Keap1 inhibitors following treatment of intact U2OS cells. Representative temperature-induced aggregation curves of Keap1-mCherry following a 3-h treatment of intact Keap1-mCherry-expressing U2OS cells with 20 µM sulforaphane, SF (A), 200 nM TBE-31 (B), 250 nM RTA-408 (C), 1 µM VVD-065 (D) (red symbols) or vehicle (0.1% DMSO, black symbols). Following compound treatment, the cells were lysed and the mCherry fluorescence intensity was measured in soluble fractions of lysates heated to 39–72 °C, normalized to that of the soluble fraction of DMSO-treated lysate heated to 39 °C.

As these experiments were conducted with cell lysates, we considered the possibility that, due to their high reactivity, the Keap1 sensor cysteine(s) could be oxidised during the lysis of the cells and thus unavailable for binding. Therefore, we next subjected intact cells to treatments with electrophiles. To ensure sufficient time for entry across the cell membrane, we increased the treatment time to 3 hours. In keeping with the results obtained with cell lysates, there was no change in the thermostability of Keap1-mCherry upon treatment with SF, TBE-31 or RTA-408 (Fig. 6A–C). We added VVD-065 to this panel of compounds because, although it also engages Cys151, the resulting conformational change in Keap1 does not weaken, but strengthens its binding to Cul3, promoting Nrf2 degradation.^24,25^ Treatment with VVD-065 did not affect the thermostability of Keap1-mCherry (Fig. 6D), similar to the Cys151-targeting Nrf2 activators SF, TBE-31 and RTA-408.

**Fig. 7:**
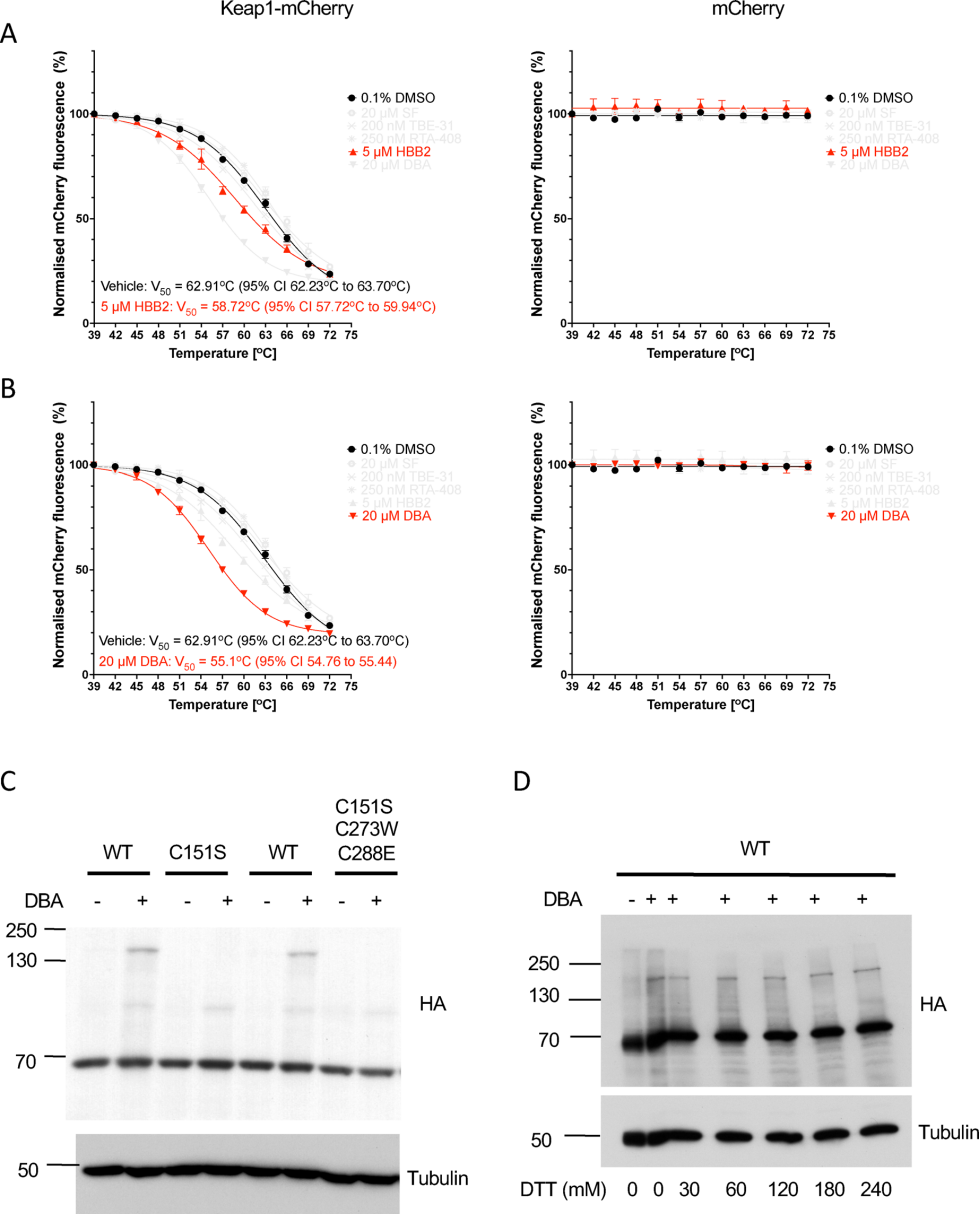
DBA treatment promotes formation of high molecular weight Keap1 species that involve Cys151. (A) and (B) Representative temperature-induced aggregation curves of Keap1-mCherry following a 3-h treatment of intact Keap1-mCherry- or mCherry-expressing U2OS cells with 5 µM HBB2 (A), 20 µM DBA (B) (red symbols) or vehicle (0.1% DMSO, black symbols). Following compound treatment, the cells were lysed and the mCherry fluorescence intensity was measured in soluble fractions of lysates heated to 39–72 °C, normalized to that of the soluble fraction of DMSO-treated lysate heated to 39 °C. (C) and (D) Immunoblotting analysis of Keap1-knockout MEFs rescued with either WT, single C151S mutant or triple C151S, C273W and C288E mutant of mouse N-terminally tagged HA-Keap1. Cells were treated with vehicle (0.1% DMSO) or DBA (20 µM) for 3 h, after which the cells were lysed. Cell lysates (15–20 µg of total protein) were subjected to SDS-PAGE either without (C) or with (D) prior incubation with increasing concentrations of DTT for 30 min at 37 °C. HA-Keap1 was detected using an antibody against HA; α-tubulin served as a loading control.

Interestingly however, treatment with HBB2 or DBA caused a decrease in the thermostability of Keap1-mCherry (but not free mCherry) (Fig. 7A and B). Since these compounds contain symmetrical Michael acceptor moieties, we considered the possibility that they may serve as a molecular bridge, promoting the formation of a Keap1 dimer. Taking into account the high reactivity of Cys151, we tested this possibility by use of Keap1-knockout mouse embryonic fibroblasts (MEFs) that had been rescued with either HA-tagged wild-type (WT) or mutant forms of Keap1, where Cys151 is substituted with a serine residue, *i.e.* a single C151S Keap1 mutant or a triple C151S, C273W and C288E Keap1 mutant.^12^ Treatment of WT MEFs with DBA caused the appearance of a slower-migrating high molecular weight species, which were detected with the HA antibody (Fig. 7C). The most prominent of these high molecular weight species was not observed in cells harbouring a C151S mutant Keap1, implying the involvement of Cys151 in its DBA-mediated formation. It was then envisioned that, due to their relatively low selectivity,^50^ compounds like HBB2 and DBA could be causing glutathione depletion and oxidative stress, which in turn causes formation of an intermolecular disulfide bridge linking two Keap1 molecules. Such high molecular weight species of Keap1 have been reported previously by Fourquet *et al.*^51^ in HeLa cells ectopically expressing HA-Keap1 and/or Myc-His-Keap1 following treatment with the oxidant hydrogen peroxide. However, incubation of lysates of DBA-treated cells with high concentrations of the reducing agent dithiothreitol (DTT) before electrophoresis had no effect (Fig. 7D). Although the precise identity of this species remains unknown, it is consistent with DBA-mediated formation of a covalent dimer of Keap1 that involves Cys151, which in turn might be responsible for the decrease in the thermostability of Keap1.

## Discussion

Binding of Keap1-Nrf2 PPI inhibitors to Keap1 has been demonstrated using a variety of *in vitro* approaches, including fluorescence polarization, isothermal calorimetry, and co-crystallization. These assays have provided valuable insights into the nature of the Keap1-PPI inhibitor binding. However, they are all conducted outside the cellular context and thus only partially reflect the complex cellular environment. This limitation has been overcome by use of the cellular thermal shift assay (CETSA), where compound binding causes a thermal shift of Keap1, which can be detected by western blotting and/or fluorescence methods without the need for highly specialised equipment or training.^27,34^ In this study, using a fluorescence-based CETSA, we show that Keap1-Nrf2 PPI inhibitors increase the thermostability of their protein target, Keap1, in lysates of cells as well as intact cells. Notably, we have only tested the concentration dependence of the thermal shift with one of the most potent compounds, KI-696. Whereas this is a limitation of our study, considering that all PPI inhibitors tested bind to the Kelch domain of Keap1, we expect a similar concentration dependence for all compounds.

One of the most interesting, and somewhat unexpected, findings of this study was the lack of increase in the thermostability of Keap1 following treatment with most of the electrophilic Nrf2 activators tested. Notably, this does not indicate absence of target engagement or biological effectiveness; indeed, all of the tested electrophiles were used at concentrations previously shown to activate Nrf2 in multiple cell lines, and in many cases, to bind to Keap1.^9,13,44,50^ Whereas at first glance surprising, this result could be explained by the fact that, although these compounds bind covalently to the cysteine sensor(s) of Keap1, this reaction is reversible, as previously demonstrated by variable temperature NMR spectroscopy studies. These studies have shown that, at elevated temperatures, the adduct formed between electrophilic Nrf2 activators and thiol nucleophiles undergo a facile decomposition back to the free electrophile.^52,53^ Thus, it is likely that although the electrophilic Nrf2 activators react readily with cysteine thiols of Keap1, the resultant adducts are unstable and rapidly undergo elimination, leading to Keap1 regeneration. This possibility is supported by our recent finding that incubation of lysates of Keap1-mCherry-expressing U2OS cells with the electrophilic metabolite of kynurenine, kynurenine-carboxyketoalkene (Kyn-CKA), which forms stable adducts with *N*-acetyl cysteine, glutathione and Keap1,^54^ does lead to thermostabilization of Keap1-mCherry.^23^ Alternatively, considering that none of the examined electrophiles bind to the Kelch domain of Keap1, whereas all of the PPI inhibitors do, it is possible that only compounds that bind to Keap1-Kelch, but not to Keap1-BTB result in alteration of the thermal stability of Keap1.

Interestingly however, HBB2 and especially DBA, both of which contain two symmetric electrophilic centers, caused a decrease in the thermostability of Keap1-mCherry upon treatment of intact cells, suggesting that such compounds have the ability to engage multiple amino acids in Keap1. This notion is further supported by the detection of a higher molecular weight species of Keap1 involving Cys151 when MEFs were treated with DBA, consistent with formation of a covalently linked Keap1 dimer. A similar double Michael acceptor, 4-ethyl-2,6-bis-pyridin-3-yl-methylene cyclohexanone (MCB-613), was recently shown to form a molecular bridge linking two Keap1 monomers *via* their BTB domains, and decrease the thermostability of Keap1.^55^ The reason why this change in the thermostability of Keap1 by HBB2 and DBA is only observed upon treatment of live cells, but not cell lysates, is currently unknown. It could be a reflection of greater electrophile availability due to its intracellular accumulation prior to conjugation with glutathione and subsequent metabolism through the mercapturic acid pathway.

**Fig. 8:**
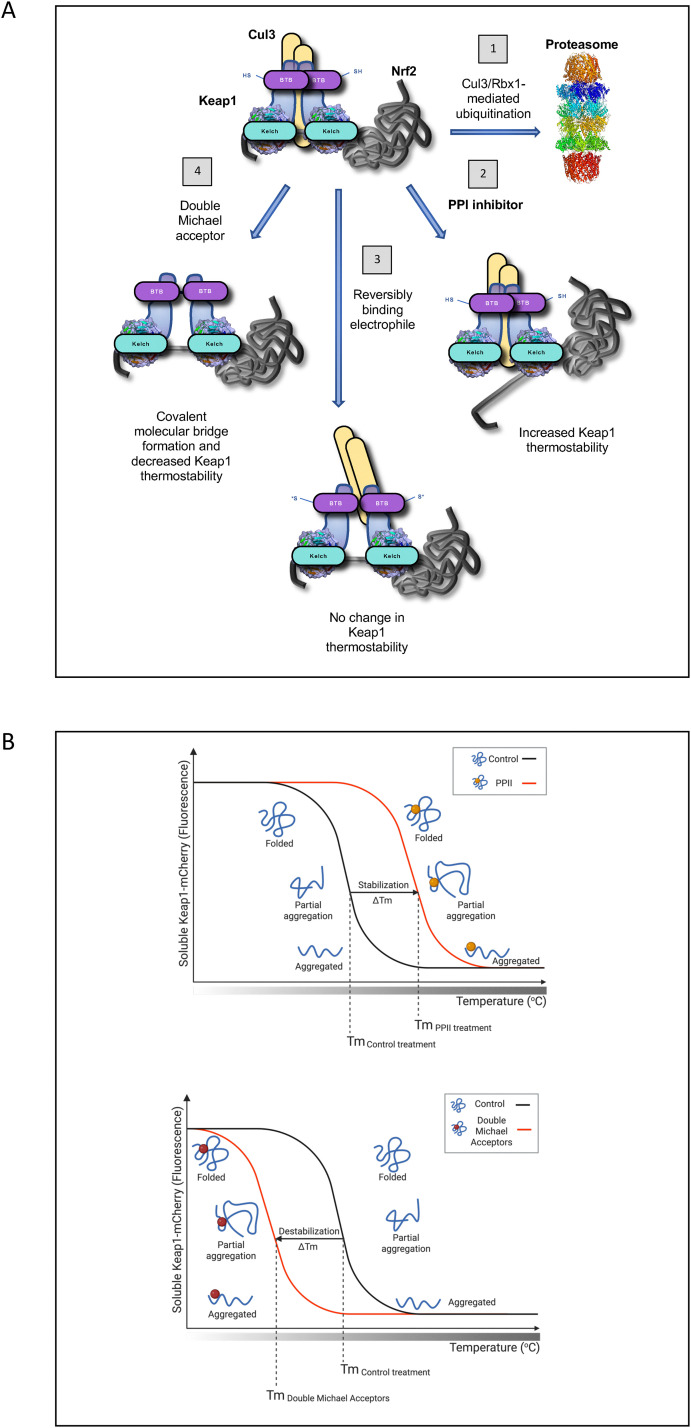
PPI inhibitors increase, whereas molecular bridge-forming double Michael acceptors decrease the thermostability of Keap1. (A) The E3 ubiquitin ligase substrate adaptor Keap1 targets transcription factor Nrf2 for Cul3/Rbx1-mediated ubiquitination and subsequent degradation through the proteasome (1). Small-molecule Keap1–Nrf2 protein–protein interaction (PPI) inhibitors bind to the Kelch domain of Keap1, causing an increase in thermostability (2). Electrophiles, which bind covalently, but reversibly to C151 in the BTB domain of Keap1, do not change the thermostability of Keap1 (3). Double Michael acceptors cause the formation of high molecular weight Keap1 species, causing a decrease in its thermostability (4). SH = reduced cysteine; S* = modified cysteine. (B) A unified Keap1 conformation alteration model upon binding to PPI inhibitors (top) and double Michael acceptors (bottom); adapted from “Thermal Shift Assay Principle” template and created in Biorender.com.

In conclusion, we show that PPI inhibitors, but not reversibly-binding electrophiles, increase the thermostability of Keap1, whereas double Michael acceptors are causing a decrease (Fig. 8). The results from this study indicate that the fluorescence-based CETSA is a convenient, inexpensive and medium-throughput option for monitoring target engagement by Keap1-targeting PPI inhibitors at physiological conditions, without the requirement for specific antibodies that are essential for immunoblot-based CETSA. Furthermore, this method can be used to distinguish between the mode of action of electrophiles that bind covalently and reversibly to Keap1 and non-covalent allosteric Keap1 inhibitors in the context of the complex cellular environment, which had previously only been possible to achieve by highly specialized, low throughput and expensive Förster resonance energy transfer (FRET)-based multiphoton fluorescence lifetime imaging microscopy (FLIM) analysis of single live cells.^35,56,57^ In addition, the fluorescence-based CETSA has the potential to be optimized for high-throughput screens and used under various experimental conditions, which would facilitate the discovery of new Keap1-Nrf2 PPI inhibitors. Its main limitation is the requirement for relatively high levels of expression of the Keap1-mCherry fusion protein, exceeding the levels of endogenous Keap1. Additionally, because the fusion of mCherry to a target protein has a thermo-stabilizing effect,^58^ it is only possible to measure relative, and not absolute, changes in thermostability upon compound binding. Furthermore, if a compound targets the C-terminus of Keap1, where the mCherry protein is fused, an effect on the thermostability of Keap1-mCherry may not be detected due to steric hindrance. Finally, as for any CETSA method in general, the possibility that a change in the thermal stability of Keap1-mCherry upon compound treatment of cells or cell lysates may not be necessarily caused by direct binding, but rather by binding to another component of the Keap1 interactome and/or triggering post-translational modification event(s), should be also carefully considered.

## Materials and methods

### Materials

Doxycycline hyclate (Dox, Sigma/Merk, D9891). Antibodies for immunoblotting: rat monoclonal anti-Keap1, clone 144 (Millipore, MABS514, 1 : 4000 dilution); anti-mCherry (Living Colors, 1 : 1000 dilution); mouse monoclonal anti-β-actin, clone AC-15 (Sigma, A 5441, 1 : 20 000 dilution); anti-rabbit, anti-rat and anti-mouse IRDye800 or IRDye-680 labelled secondary antibodies (Li-COR Biosciences, 1 : 15 000 dilution). Commercially obtained inducers were of the highest purity available: *R*,*S*-sulforaphane (SF) was purchased from LKT Labs, DBA was from Sigma, and KI-696 and RTA-408 were from MedChemExpress. PRL-295, TBE-31, HBB2, NG-316 and NG-284 were synthesized as described previously.^31,32,59,60^ VVD-065 was a generous gift from Vividion Therapeutics. All compounds were used at concentrations which had been shown previously to activate Nrf2 and induce the Nrf2-transcriptional target NQO1 in cell-based assays.

### Plasmids

pcDNA5-FRT/TO and pOG44 are from ThermoFisher. Plasmids encoding mouse Keap1-mCherry (Keap1-12fl-mCherry) and sfGFP-Nrf2 were described previously.^35^ To generate Keap1-12fl-mCherry or mCherry in pcDNA5-FRT-TO, Keap1-12fl-mCherry or mCherry-encoding fragments were excised from the above Keap1-mCherry plasmid and inserted between KpnI and NotI sites of pcDNA5-FRT-TO.

### Generation of inducible Keap1-mCherry-expressing U2OS and HeLa cell lines

Integration of Keap1-12fl-mCherry or mCherry into Flp-InTM T-RExTM host cells U2OS-FRT-TO (kind gift of Dr Laureano de la Vega) and HeLa-FRT-TO (kind gift of Prof Stephen Taylor) was done by co-transfecting cells with 1 : 1 mixture of Keap1-12fl-mCherry or mCherry in pcDNA5-FRT-TO vector and pOG44 plasmid encoding Flip recombinase, using Lipofectamine 2000 (Invitrogen), followed by selection of integrated cells under 250 µg ml^−1^ hygromycin B (ThermoFisher Scientific). Cells were further maintained in DMEM media containing 10% heat-inactivated Fetal Bovine Serum and 250 µg ml^−1^ hygromycin B. To induce expression of integrated constructs, 1 µg mL^−1^ Dox was added to the culture media for more than 20 h.

### Immunoblotting

Cells were grown on 6-well plates, washed two times with PBS and lysed in SDS lysis buffer containing 2% SDS, 62.5 mM TrisHCl pH 6.8, 10% glycerol and protease inhibitors (cOmplete Mini, EDTA-free protease inhibitor cocktail, Roche). Lysates were boiled for 2 min, sonicated and supplemented with DTT up to 100 µM and with Bromophenol Blue. 10 µg lysates were separated on 4–12% pre-cast BisTris NuPAGE gels (Invitrogen) under MOPS running buffer or a 8–10% SDS-PAGE/Tris-Glycine gel running system (Biorad) with a PageRulerTM protein ladder (ThermoFisher Scientific, 26616) and transferred onto AmershamTM Protran® Premium 0.45 µm nitrocellulose membrane under transfer buffer containing 25 mM Tris, 129 mM glycine and 20% methanol. Membranes were stained with 1% Ponceau S in 5% acetic acid, de-stained and blocked in 5% non-fat milk in PBST (PBS supplemented with 0.1% Tween 20) for 1 h before blotting with primary antibodies in blocking solution overnight at 4 °C or for 2 h at room temperature. Membranes were washed in PBST, incubated with respective secondary antibodies in blocking solution for 1 h at room temperature, washed and scanned on Odyssey CLx Infrared Imaging System or developed using the chemiluminescence system and a X-ray film developer (FUJIFILM).

### Imaging

Cells were grown in 8-well Ibidi chambers in DMEM media supplemented with 10% Fetal Bovine Serum and 1 µg ml^−1^ Dox for over 24 h and imaged using Leica DMi8 inverted microscope operated by Las X software (Leica), with HC PL APO CORR 40×/0.85 objective and 540–580 excitation/592–668 emission filters (for mCherry) or transmitted light with DIC filter. Images were acquired and processed in ImageJ/FIJI identically.

### Measuring Nrf2-Keap1 interaction by FLIM-FRET

HeLa-FRT-TO cells with integrated Keap1-12fl-mCherry or mCherry were seeded into 3.5 cm glass-bottom FluoroDishes (World Precision Instruments) with or without 1 µg ml^−1^ Dox, at 2 × 10^5^ cells per dish, a day before transient transfection of sfGFP-Nrf2 plasmid using the calcium phosphate method as described.^36^ One day later, sfGFP fluorescence lifetime was measured as described^36^ using SPC-150 Time-Correlated Single Photon Counter (TCSPC) module (Becker&Hickl) attached to LSM 710 microscope (Zeiss) with 2-photon Chameleon laser (Coherent) and HPM-100-40 GaAsP detector. Fluorescence Lifetime Imaging (FLIM) data acquisition was operated by SPCM software (Becker&Hickl) controlling TCSPC module, while laser was controlled by Zen software (Zeiss). sfGFP fluorescence lifetime was measured at 920 nm and with GFP filters. Data were analysed in SPCImage (Becker&Hickle) using 1-component exponential decay fitting in the outlined cytoplasmic areas of the cells. Plots were produced using R.

### CETSA

CETSA was performed according to a previously described protocol.^34^ The expression of Keap1-mCherry or mCherry was induced in U2OS-FRT-TO and HeLa-FRT-TO cells (2 × 10^6^ cells per 15 cm dish) with integrated Keap1-12fl-mCherry or mCherry by treatment (24 hours post-seeding) with 0.5 µg ml^−1^ of doxycycline hyclate for 48 h to 96 h. The induced cells were collected in 1 mL PBS supplemented with protease inhibitor cocktail (Roche) (PBS-PIC), flash frozen in liquid N_2_, and stored at −80 °C. The cells were thawed and lysed by 5–6 freeze–thaw cycles. Clear lysates were obtained following centrifugation at 9600× *g* for 5 min and incubated with each compound or vehicle (0.1% DMSO) for 1 h at 37 °C. After incubation, the lysates from each treatment were aliquoted (100 µL) into 12 PCR tubes (I1402-3700, Starlab) heated for 3 min at the indicated range of temperatures and let to cool at 25 °C for a further 3 min (Veriti Thermal Cycler, Thermo) before placing on ice. For treatments of intact cells, cells were incubated with each compound or vehicle (0.1% DMSO) for 3 h at 37 °C in serum-free media, washed thrice with PBS, collected in 1 ml of PBS-PIC and subsequently flash frozen in liquid N_2_, and stored at −80°. Subsequently, the cells were freeze-thawed by 5–6 cycles. The lysates were clarified by centrifugation at 9600 × *g*, and heated for 3 min at the indicated range of temperatures and let to cool at 25 °C for a further 3 min (Veriti Thermal Cycler, Thermo). In all experiments, the total protein concentration in the lysates was between 1–2 mg ml^−1^.

The aggregated material was removed by a high-speed centrifugation at 15 000× *g* at 4 °C for 60 min (5810R, Eppendorf). After centrifugation, the 80 µL soluble fraction (supernatant) was very carefully pipetted and transferred to an opaque white plate (Thermo). The mCherry fluorescence of the soluble fraction (supernatant) was measured using the TECAN SPARK plate reader (TECAN) with 580 nm excitation (20-nm excitation bandwidth) and 635 nm emission (35-nm emission bandwith) wavelengths. Each experiment was performed at least three times. Each point plotted on the graphs shows the mean −/+ the standard error of the mean (SEM). In each figure, the control curve (0.1% DMSO) was generated by pooling all the data points from the various independent experiments to generate a single curve. The curve for each indicated compound is represented in red, whilst the curves for all the other compounds are greyed.

For observing the isothermal dose–response (ITDR) and generating the ITDR curve,^61^ lysates of Keap1-mCherry-expressing U2OS cells (*n* = 3) were aliquoted (100 µL) and incubated with a range of concentrations of KI-696 or vehicle (0.1% DMSO) for 1 h at 37 °C. Following incubation, the lysates were subjected to heat treatment at 62 °C for 3 min, and subsequently cooled to 25 °C for a further 3 min. The aggregated material was removed by centrifugation and the Keap1-mCherry fluorescence in the soluble fraction was measured as described above. The ITDR curve was generated by plotting the fluorescence intensities of the soluble fractions each of the concentrations tested relative to the fluorescence intensity of the soluble fraction of the vehicle (0.1% DMSO) condition and represented as a percentage.

### Immunoblotting of Keap1

The generation of stable Keap1-knockout (KKO) Keap1-rescued MEF cell lines has been described previously.^12^ Briefly, immortalized Keap1-knockout (KKO) MEFs were cultured at 37 °C and 5% CO_2_ in low glucose Dulbecco modified Eagle medium (DMEM, Wako Chemical, Japan) supplemented with 9% FBS. Stable cell lines expressing HA (amino acid sequence: YPYDVPDYA)-tagged mouse Keap1 (wild-type HA-Keap1), the single C151S mutant of HA-Keap1, or the triple C151S, C273W and C288E mutant of HA-Keap1 were obtained by electroporation of the cDNA encoding the corresponding protein sequence using the PiggyBac transposon system (PB514B-2; System Biosciences, California, USA) followed by puromycin (2 mg ml^−1^) selection. Single colonies were isolated, expanded, and the relative expression of HA-Keap1 was determined in whole cell lysates by immunoblotting using rat monoclonal HA antibody (1 : 1000 dilution; Roche, 3F10, California, USA) and the α-tubulin antibody (DM1A clone, Sigma-Aldrich).^12^

### Data quantification and statistical analysis

The FLIM data were analysed in SPCImage (Becker&Hickle) using 1-component exponential decay fitting in the outlined cytoplasmic areas of the cells. For CETSA, the fluorescence intensity for each treatment was normalised to the fluorescence intensity of 39 °C and set as a percentage relative to that temperature. GraphPad Prism 10 was used to generate the nonlinear thermal shift curves using the Boltzmann sigmoidal function where the top of the curve was constrained to 100. The unconstrained *V*_50_ values for each curve are obtained within the ‘Table of Results’ generated by this function along with the 95% confidence intervals (CI) of the *V*_50_. The *V*_50_ value represents the midpoint value between the top plateau and the bottom value of the curve that has been fitted, which can also be interpreted as *T*_m_, or melting temperature. The 95% confidence interval of the fitted *V*_50_ is stated for each of the curves in the figures.

## Author contributions

S. D. N. optimized the cellular thermal shift assay (CETSA), designed, planned and performed the biochemical and biological experiments in all cell lines, and participated in writing the paper. D. D. created and validated the Keap1-mCherry- and mCherry-expressing cell lines, developed fluorescence-based CETSA and performed the initial CETSA experiments in these cell lines, and participated in writing the paper. J. M. W., C. J. Y. L., A. J. C., M. T. and J. F. performed CETSA experiments during the early stages of the project. T. W. M., T. J., T. H. and G. W. synthesized and provided compounds. T. S. and M. Y. provided the MEF cells and helped plan the experiments. A. T. D.-K. planned and administered the project, and wrote the manuscript with input from S. DN and D. D.

## Conflicts of interest

The work of the A.T. D.-K. lab has received funding from Reata Pharmaceuticals/Biogen. None of the authors have any known competing financial interests or personal relationships that could have appeared to influence this work.

## Supplementary Material

CB-007-D6CB00045B-s001

## Data Availability

The data supporting the findings in this article are included in the main text and figures. The raw values from the plate reader will be made available upon reasonable request from the authors. The raw images of the immunoblots are in the supplementary information (SI). Supplementary information is available. See DOI: https://doi.org/10.1039/d6cb00045b.
