# Supplementary material for: Keap1-Kelch-targeting protein–protein interaction inhibitors, but not reversibly-binding electrophiles, increase the thermostability of Keap1 in the cellular environment
Source: RSC Chem Biol. 2026 Apr 7;7(5):906–22. doi: 10.1039/d6cb00045b (PMC13093462; doi:10.1039/d6cb00045b)
Supplement: CB-007-D6CB00045B-s001 [file CB-007-D6CB00045B-s001.pdf]

Supplementary Information (SI) for RSC Chemical Biology.
This journal is © The Royal Society of Chemistry 2026

Supplementary Information

Keap1-Kelch-targeting protein-protein interaction inhibitors, but not reversibly-binding electrophiles, increase the thermostability of Keap1 in the cellular environment

Sharadha Dayalan Naidu, Dina Dikovskaya, Jasmine M. Walker, Charlotte Lim Jia Yee, Annamarie J. Cafferkey, Manaka Tatsuno, Jialin Feng, Terry W. Moore, Tatum Johnson, Tadashi Honda, Geoff Wells, Takafumi Suzuki, Masayuki Yamamoto, Albena T. Dinkova-Kostova

Raw images of the immunoblots shown in Figure 7

Figure 7C

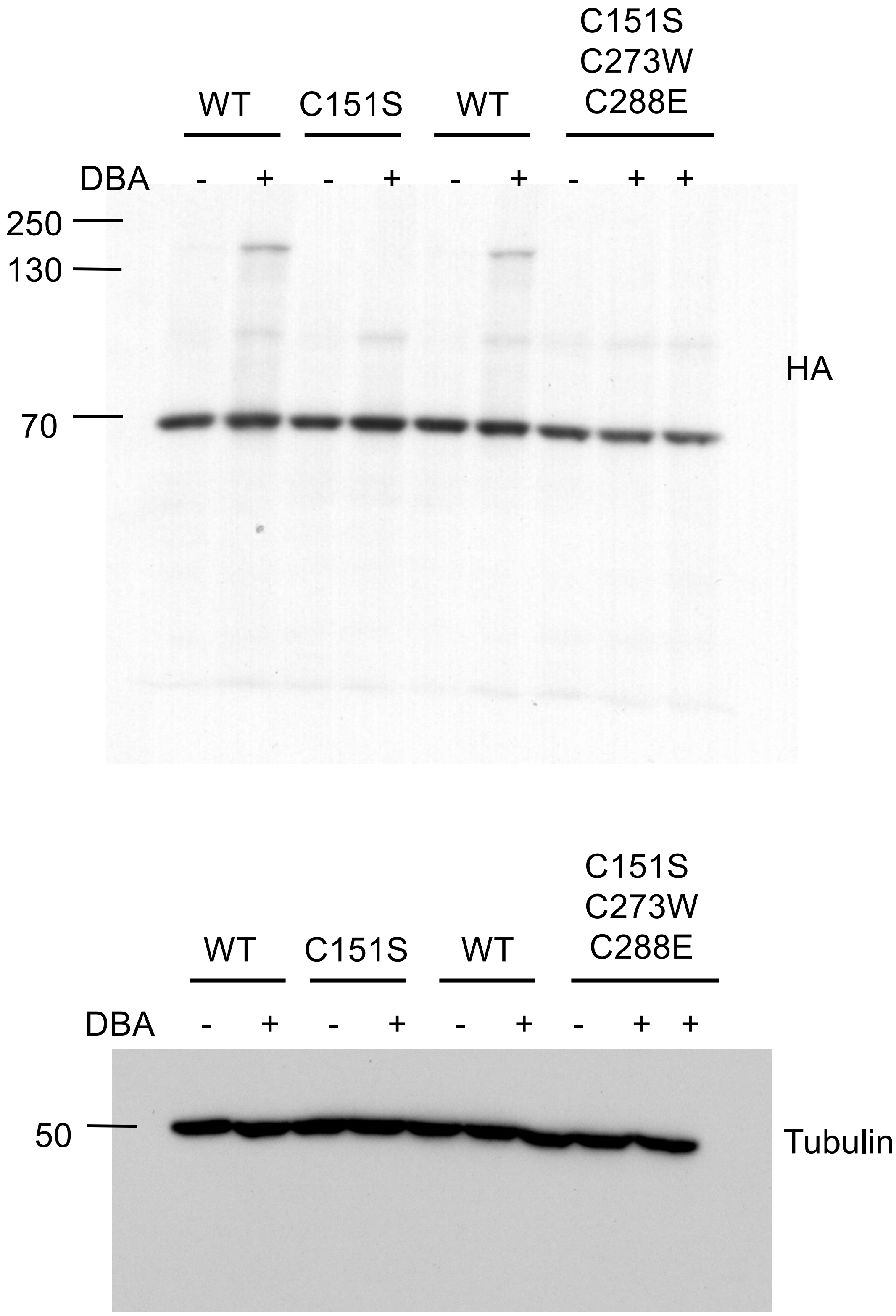

WT
C151S
WT
C151S
C273W
C288E
DBA
- + - + - + - + +
250
130
70
HA
WT
C151S
WT
C151S
C273W
C288E
DBA
- + - + - + - + +
50
Tubulin

Figure 7D

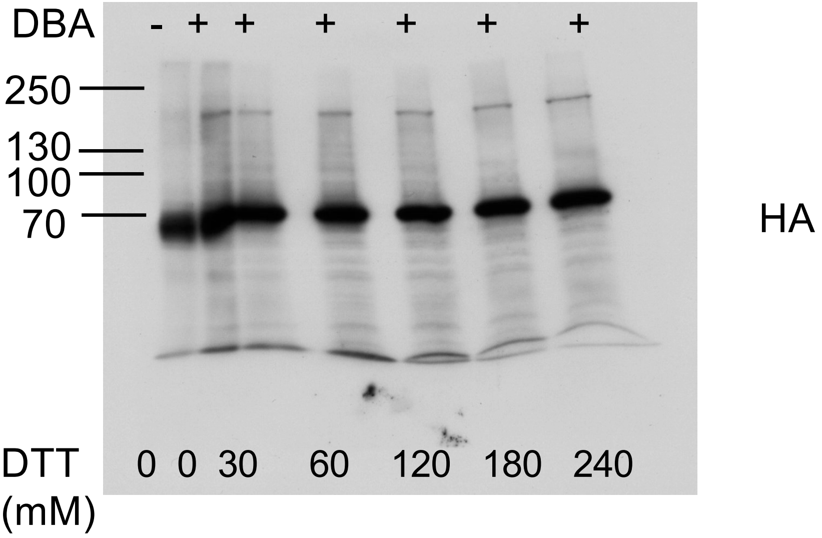
DBA - + + + + + +
250
130
100
70
HA
DTT 0 0 30 60 120 180 240
(mM)

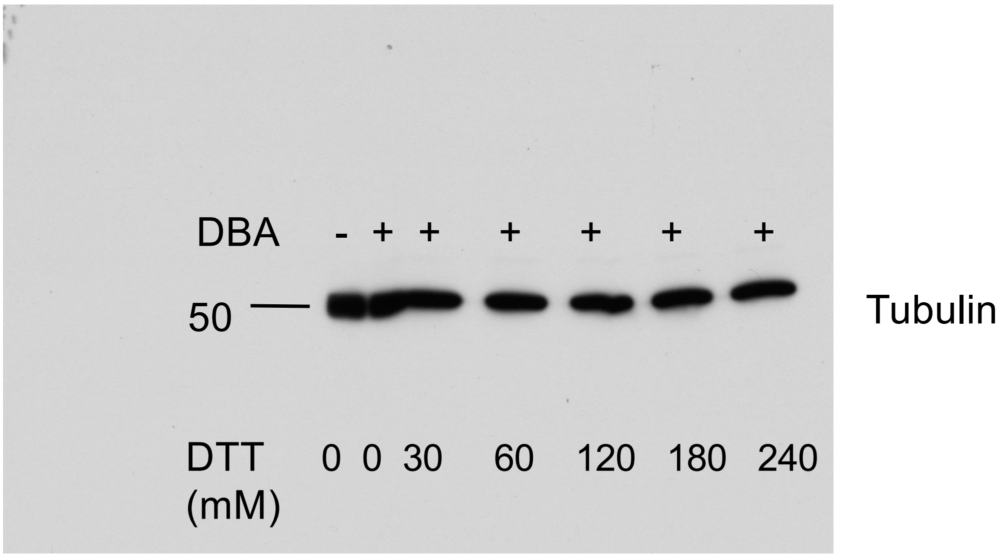
DBA - + + + + + +
50
Tubulin
DTT 0 0 30 60 120 180 240
(mM)
